# Task shifting in primary care to tackle healthcare worker shortages: An umbrella review

**DOI:** 10.1080/13814788.2021.1954616

**Published:** 2021-08-02

**Authors:** Siew Lian Leong, Siew Li Teoh, Weng Hong Fun, Shaun Wen Huey Lee

**Affiliations:** a Faculty of Pharmacy, Cyberjaya University College of Medical Sciences, Selangor, Malaysia; b School of Pharmacy, Monash University Malaysia, Selangor, Malaysia; c Institute for Health Systems Research, National Institutes of Health, Shah Alam, Malaysia; d Asian Centre for Evidence Synthesis in Population, Implementation and Clinical Outcomes (PICO) Health and Well-being Cluster, Global Asia in the 21^st^ Century (GA21) platform, Monash University, Bandar Sunway, Malaysia; e Faculty of Health & Medical Sciences, School of Pharmacy, Taylor’s University, Selangor, Malaysia

**Keywords:** Umbrella review, primary care, task shift, health care organisation and systems, barriers and facilitators

## Abstract

**Background:**

Task shifting is an approach to help address the shortage of healthcare workers through reallocating human resources but its impact on primary care is unclear.

**Objectives:**

To provide an overview of reviews describing task shifts from physicians to allied healthcare workers in primary care and its impact on clinical outcomes.

**Methods:**

Six electronic databases were searched up to 15 December 2020, to identify reviews describing task shifting in primary care. Two reviewers independently screened the references for relevant studies, extracted the data and assessed the methodological quality of included reviews using AMSTAR-2.

**Results:**

Twenty-one reviews that described task shifting in primary care were included. Task shifted include provision of care for people with chronic conditions, medication prescribing, and health education. We found that task shifting could potentially improve several health outcomes such as blood pressure, HbA1c, and mental health while achieving cost savings. Key elements for successful implementation of task shifting include collaboration among all parties, a system for coordinated care, provider empowerment, patient preference, shared decision making, training and competency, supportive organisation system, clear process outcome, and financing.

**Conclusion:**

Evidence suggests that allied healthcare workers such as pharmacists and nurses can potentially undertake substantially expanded roles to support physicians in primary care in response to the changing health service demand. Tasks include providing care to patients, independent prescribing, counselling and education, with comparable quality of care.

KEY MESSAGES

- Task shift from physicians to allied healthcare professionals in primary care appears to increase service provision and cost-effectiveness.
- Services shifted include the provision of care and coordination of patients with chronic diseases as well as independent prescribing.
- This requires optimisation of organisation systems, engaging with all stakeholders to ensure health systems strengthening.

## Introduction

Globally, the world is ageing with more people living longer [1]. Therefore, healthcare systems need to adapt and adjust in meeting the rising demand for quality healthcare of this population. A good primary care system plays an essential role, as it provides quality people-centred healthcare services for the ageing population close to their homes. Unfortunately, human resource in health is limited, especially physicians working in primary care. Currently, only two in every five countries worldwide meets the World Health Organisation’s (WHO) minimum recommendation of physician to population ratio of 1:1000 [2–4].

Task shifting involves the rational redistribution of tasks to individuals within the healthcare team with fewer qualifications that conventionally were not within their scope of work [5,6]. This management technique has been advocated as an important strategy to optimise health system performance, especially in resource poor settings. Studies performed to date have shown that task shifting can address healthcare resource shortages and allow physicians in primary care to provide more complex care and expand the healthcare capacity [7–9].

This concept was first developed as a strategy to provide care for individuals with HIV in sub-Saharan Africa where there was shortage of specialised healthcare workers [10,11], due to the disparity between healthcare services, capacity, and budget. In response to this, the WHO developed a consolidated guideline on using task shifting to tackle health worker shortages [5]. Since then, this concept has been expanded to other disease states such as mental health as well as expanded services, including pharmacist-led warfarin clinics. These substitutions are strategies to improve access, efficiency and quality of care in many countries, especially in low- and middle-income countries. Indeed, expanding the roles of allied healthcare workers have been advocated as one of the strategies to enhance the quality of care towards achieving the Sustainable Development Goal 3 of maintaining good health and well-being [12].

## New contribution

Multiple systematic reviews on task shifting to other healthcare workers, including nurses and pharmacist have been published [13–15]. However, to our best knowledge, there is no comprehensive overview of systematic reviews on task shifting that examined the various roles and responsibilities of allied health workers in primary care and how these strategies can be implemented optimally. Such evidence is vital for health policy planning, especially in resource poor settings and pandemics such as COVID-19, where healthcare resources are strained. Umbrella reviews are reviews of existing systematic reviews that summarise the evidence from multiple systematic reviews and meta-analyses on the same topic [16–18]. The evidence generated is derived from the highest level of evidence, i.e. from systematic reviews and meta-analysis into one single article which provides quality evidence for policy decision-making. In this umbrella review, we had two objectives, namely: (1) to describe the types of interventions or task that were shifted from physicians to other allied healthcare workers in primary care and (2) to describe the impact of task shifting on any clinical outcomes.

## Methods

We followed the PRISMA reporting guideline [19]. The study was registered with PROSPERO (Registration no: 180151).

### Search strategy

A comprehensive literature search was performed on MEDLINE, EMBASE, CINAHL, AMED, PsycINFO and the Cochrane Library from database inception to 15 April 2020, for systematic reviews investigating task shifts to allied healthcare professionals in primary care. We updated our search to 15 December 2020, upon peer review. This was supplemented with a grey literature search on PROSPERO registry and hand search of identified articles without language restriction. Search keywords used included a combination of terms related to task shifting, delegation, allied health workers and primary care. A complete list of keywords used can be found in Supplementary Box S1.

### Inclusion/exclusion criteria

Systematic reviews with or without meta-analyses that examined allied health workers engaged in task shifting activities in primary care were eligible for inclusion. This study only examined five allied health groups including nurses, pharmacists, dieticians, physiotherapists, and paramedics. This was because studies have shown a synergy between the roles of these individuals with the healthcare systems and patient contact points [20,21]. These task shifting roles could encompass worker substitution or delegation across any disease or medical condition. A study was considered as systematic review if it described the conduct of review in sufficient detail and attempted to identify all relevant primary studies using at least one database, with a search strategy provided. Studies were excluded if these were primary studies, narrative reviews, literature reviews or had examined roles of other health workers such as traditional healers, social workers and community health workers.

### Data extraction and quality assessment

Two authors (SWHL and SLT) independently screened the titles and abstracts for eligibility. Full texts of relevant articles were retrieved and independently scrutinised by two authors for eligibility. Any discrepancies were resolved by a third reviewer (SLL). Data was independently extracted by two authors (SLL and SLT) and verified by a third author (SWHL). For each review, the following data was extracted first author, publication year, task shift involved, allied health professional, roles, number of studies, study design of primary studies and impact of task shift on clinical parameters. These could include but not limited to blood pressure control, glycaemic control, drug adherence, access to care of a healthcare facility and cost-effectiveness as described by each individual study. For reviews that included meta-analyses, we extracted the effect size (relative risk, odds ratio, hazard ratio or standardised mean difference) with their corresponding 95% confidence intervals. The methodological quality of included reviews was evaluated using the AMSTAR-2 tool [22] and compliance with PRISMA guidelines.

### Data synthesis

As most systematic reviews included both quantitative and qualitative studies, a narrative synthesis approach was used. All the studies were grouped according to the allied healthcare workers job function together and services that were task shifted. We then summarised the evidence for task shifting based upon guidelines by the Joanna Briggs Institute [23].

## Results

### Literature search

**Figure 1.**
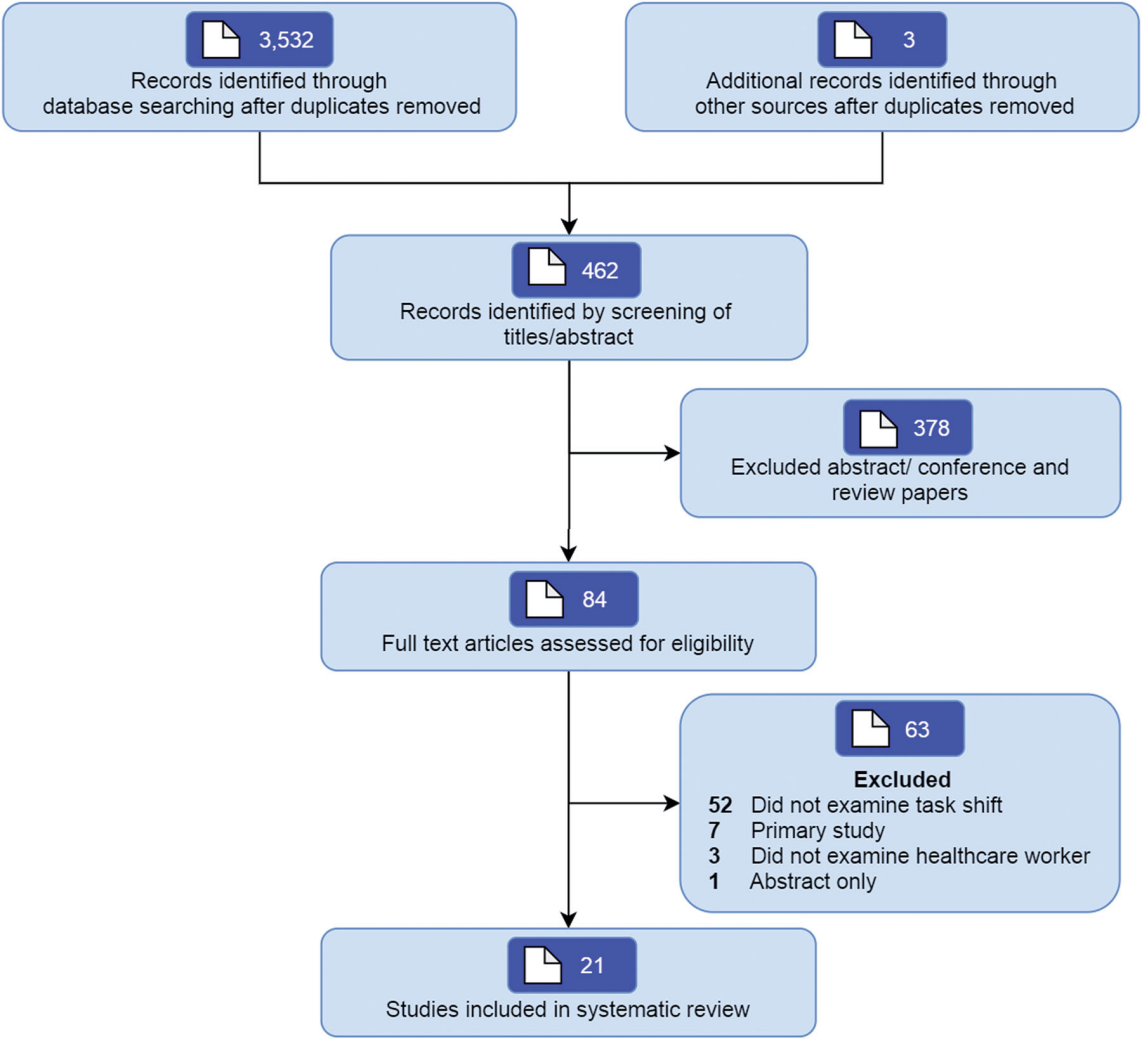
Study identified and included into the current review.

The search identified a total of 3535 articles of which 84 studies were selected for further review. A total of 63 studies were excluded as they did not examine task shift (*n* = 52), were primary studies (*n* = 7), did not examine task shift to healthcare workers (*n* = 3) or was published as an abstract (*n* = 1). Twenty-one unique reviews were finally included (Figure 1).

### Characteristics of included reviews

Most reviews included studies from high-income countries such as United States, United Kingdom, the Netherlands, Russia, Canada and Australia. Only two reviews specifically examined task shifting in low-and-middle-income countries [24,25]. Sixteen reviews summarised data quantitatively and nine included meta-analyses (Supplementary Tables S1–S3). We also identified four reviews describing the cost and cost-effectiveness of task shifting [26–29]. Full description of the task shifts is described below.

### Task shifting

####  

##### Task shifting to nurses

Fifteen reviews described task shifts from physicians to nurses [13–15,24–27,29–36]. Tasks shifted included caring for patients with chronic diseases such as hypertension and diabetes, initiation and monitoring of patients with HIV/AIDS on antiretroviral therapy (ART) [14,24,31,34], as well as medication prescribing using an algorithm [13]. Several reviews also described how nurses took on the role of case managers to deliver care, monitor and manage diseases based upon clinical guidelines and validated tools [27,30,33,35,36]. We also found reviews describing task shifts of more complex procedures such as abortions [37] and care of people with mental health [25] to nurses (Table 1).

**Table 1.** Details of studies describing task shift to nurses in primary care.

Author, year	Setting	Evidence reviewed	Task shift	Conclusions
Anthony, 2019 [26]	Primary care	Economic evaluation studies	Role substitution of work (any) that was previously completed by a GP to nurses in primary care.	Nurse-led care for common minor health problems was as effective and less costly than GP care. However, this is reliant on salary differences in the setting.
Chapman, 2004 [36]	Primary care	RCTs, analytical intervention, and observational studies	Improving access to primary care by recent innovations in the United Kingdom, including nurse-led telephone consultations in general practice and nurse-practitioner led care.	There appears to be improved access to primary care through diversification of care provision to nurses, which was as safe and effective as care by GPs.
Karimi-Shahanjarini, 2019 [15]	Primary care	Qualitative Studies	Doctor-nurse substitution including preventive care, follow-up, health promotion, maternity care as well as acute and chronic care such as diabetes, dementia care and wound care.	There is limited understanding of nurses’ role among patients, and differences between nurse-led and doctor-led care. Patients generally preferred doctors when task was considered more ‘medical’ but may accept the use of nurses to deliver services that are more health promotive or preventive in nature.
Kredo, 2014 [34]	Primary care	RCTs and observational studies	Physician to nurse substitution to either initiate and maintain ART or nurses follow up patients previously initiated on ART by doctors, for maintenance care of ART.	Some evidence suggest shifting responsibility from doctors to adequately trained and supported nurses for managing patients probably does not decrease the quality of care and, in the case of nurse-initiated care, may decrease the numbers of patients lost to follow-up.
Laurant, 2018 [14]	Primary care	RCTs	Physician-nurse substitution of roles and services in primary care except for mental health problems.	Nurse-led primary care may lead to fewer death in diseases such as cardiovascular care, diabetes and rheumatic diseases. Consultation time was longer but they also had better patient return rates.
Martinez-Gonzalez, 2014 [31]	Primary care	RCTs	Physician-nurse substitution to provide care for complex conditions including HIV, hypertension, heart failure, cerebrovascular diseases, diabetes, asthma, Parkinson’s disease, incontinence, mental health and addiction.	Meta-analyses showed greater reductions in systolic blood pressure in favour of nurse-led care (WMD −4.27 mmHg, 95% CI-6.31 to −2.23) but not diastolic blood pressure (WMD −1.48 mmHg, 95%CI −3.05 to −0.09), total cholesterol (WMD −0.08 mmol/L, 95%CI −0.22 to 0.07) or glycosylated haemoglobin (WMD 0.12%HbAc1, 95%CI −0.13 to 0.37). Of other 32 clinical parameters identified, less than a fifth favoured nurse-led care while 25 showed no significant differences between groups.
Martinez-Gonzalez, 2014 [30]	Primary care	RCTs and economic evaluation studies	Nurses (in any type of role) substituted physicians as case manager and could delegate clinical responsibility for tasks that were formerly performed by physicians’ alone.	Patients were generally more satisfied with nurse-led care (SMD 0.18, 95% CI 0.13–0.23). Nurse-led care was effective at reducing the overall risk of hospital admission (RR 0.76, 95% CI 0.64–0.91) and mortality (RR 0.89, 95% CI 0.84–0.96).
Martinez-Gonzalez, 2015 [32]	Primary care	RCTs	Task shifting of care from family physicians, paediatricians and/or geriatricians to nurses in all roles.	No differences in the quality of care provided by nurses and physicians were noted. Patients who received nurse-led care achieved better outcomes in the secondary prevention of heart disease and a greater positive effect in managing dyspepsia and at lowering cardiovascular risk in diabetic patients.
Martinez-Gonzalez, 2015 [33]	Primary care	RCTs	Physician-led care (family physicians, paediatricians and geriatricians) to nurse-led care (all nurse roles) based on a substitution model.	Trained nurses could provide care that was at least equivalent to those provided by physicians in the management of chronic diseases such as hypertension, asthma and obesity management. Other potential roles that could be shifted to nurses with favourable outcomes include health education and promotion.
Martinez-Gonzalez, 2015 [27]	Primary care	RCTs	Physician-led care (family physicians, paediatricians and geriatricians) to nurse-led care (all nurse roles) based on a substitution model.	Task shifting to nurses could effectively improve patients’ return rate for consultations (OR: 1.22, 95% CI: 1.09–1.37) and was cost effective. However, this needs to come hand-in-hand with having access to resources, including staffs, equipment and supplies, quality leadership and a sound referral system.
Ogedegbe, 2014 [24]	Primary care in low-middle income countries	RCTs	Physician to nurse substitution for medication prescribing, medication adjustment, home visits and health education among individuals with hypertension and diabetes.	Some evidence of improvement in blood pressure and glycated haemoglobin among care recipients.
Rashid, 2010 [35]	Primary care	Qualitative studies	Doctor-nurse substitution on clinical roles traditionally performed by doctors, including minor ailments and pain management.	Work delegation to nurses provided a means of organising workload within a practice. Patients generally felt nurses were able to deal with simple conditions but preferred to consult with a general practitioner for more ‘complex’ conditions due to concerns over nurses’ knowledge base, particularly in diagnostics and therapeutics, and their levels of training and competence.
van Ginneken, 2013 [25]	Primary care in low-middle income countries	RCTs and observational studies	Specialist-nurse substitution to provide care for people with mental, neurological and substance-use disorders.	The introduction of nurse achieved similar outcomes in reducing perinatal depression, severity of mental disorder, carer burden and re-admission rates compared to usual care. Nurse-led intervention could also reduce the amount of alcohol consumed among those with alcohol issues.
Weeks, 2016 [13]	Primary and secondary care	RCTs, controlled before-and-after studies and interrupted time series analysis	Non-medical prescribing by nurses versus medical prescribing for acute and chronic disease management.	After training, nurses could prescribe medications and manage a range of chronic conditions with comparable outcomes to doctors.
Whiteford, 2016 [29]	Primary care	RCTs	Doctor nurse substitution to care for patients with chronic ear, nose and throat complaints.	Studies indicated a higher level of patient satisfaction, cost benefits and lower levels of pain/discomfort in nurse-led clinics.

ART: antiretroviral therapy; MD: mean difference; OR: odds ratio; RCT: randomised controlled trials RR: relative risk; SMD: standardised mean difference; WMD: weighted mean difference.

##### Task shifting to pharmacists

Seven reviews described task shifts to pharmacists [13,26,28,36,38–40], who played the role of independent prescribers, expanding their responsibility in managing patients with chronic diseases in an attempt to relieve the burden of physicians and primary care (Table 2). Unlike nurses [30,32], pharmacist prescribing was reported to be more autonomous, with reliance on clinical judgement and guidelines. One review also described the role of community pharmacists as a suitable alternative to general practice consultations for management of minor ailments such as diarrhoea, head lice or cough and improves access to healthcare services (Table 2).

**Table 2.** Details of studies describing task shift to pharmacists in primary care.

Author, year	Setting	Evidence reviewed	Task shift	Conclusions
Anthony, 2019 [26]	Primary care	Economic evaluation studies	Role substitution of work (any) that was previously completed by a GP to pharmacists in primary care.	Pharmacist-led services for medicines management of coronary heart diseases were as effective as, but more costly than GP care. Management of chronic pain by pharmacists was more effective but more costly than GP care.
Chapman, 2004 [36]	Primary care	RCTs, analytical intervention, and observational studies	Improving access to primary care through pharmacist-led initiatives.	There was weak evidence that pharmacists can manage patients. Evidence supports pharmacist’s role in treatment of minor ailments using over-the-counter medication with low rates of onward referral to GPs.
Jebara, 2018 [38]	Community pharmacy	Surveys and qualitative studies	Independent pharmacists prescribing whereby pharmacists are permitted to assume professional responsibility for performing patient assessments; ordering drug therapy‐related laboratory tests; administering drugs; selecting, initiating, monitoring, continuing, and adjusting drug regimens.	There were positive views and experience on independent pharmacist prescribing from various group of stakeholders. Regardless of the implementation stage, benefits on ease of patient access to healthcare services, improved patient outcomes and reduced physician workload were reported. In addition, pharmacists reported empowerment due to better use of skills and knowledge and improved job satisfaction. Nevertheless, organisational issues related to financial support, role recognition and access to patient clinical records need further attention to ensure success and sustainability.
Nkansah, 2011 [39]	Primary care	RCTs	Physician to pharmacist substitution for management of drug therapy, including prescribing and modifying medication for hypertension.	Patients achieved better blood pressure control when managed by a pharmacist compared to physician.
Paudyal, 2011 [28]	Community pharmacy	RCTs and observational studies	Shifting care for minor ailments care from physicians to community pharmacists.	Community pharmacists-led minor ailment scheme was effective as there was a low re-consultation rate and high symptom resolution suggesting minor ailments were dealt appropriately. These consultations were less expensive than GP consultation.
Weeks, 2016 [13]	Primary and secondary care	RCTs, controlled before-and-after studies and interrupted time series analysis	Non-medical prescribing by pharmacist versus medical prescribing for acute and chronic disease management.	Non-medical prescribing by pharmacists who had a high degree of autonomy and collaborative support can deliver comparable outcomes to usual medical care prescribing by doctors.
Zhou, 2019 [40]	Primary care	Qualitative studies	Prescribing delegation from GPs to pharmacists, either supplementary, independent or collaboratively.	Several identified barriers to pharmacist prescribing include inadequate training, support from stakeholders and funding/ reimbursement. These studies highlight the importance of fostering a favourable socio-political context and prescriber competence through clear policy pathways, targeted training courses, raising stakeholder recognition and identifying specific funding, infrastructure and other resourcing.

RCT: randomised controlled trials.

##### Task shifting to other healthcare professionals

Four reviews described how healthcare workers such as midwife and community healthcare workers took on additional roles, including provision of abortion procedures, telephone consultation, medication prescribing and health education promotion compared to physicians (Table 3) [24,26,37,41].

**Table 3.** Details of studies describing task shift to other allied healthcare workers in primary care.

Author, year	Setting	Evidence reviewed	Task shift	Conclusions
Anthony, 2019 [26]	Primary care	Economic evaluation studies	Role substitution of work (any) that was previously completed by a GP to community health practitioners.	Task shifting and role substitution by community health practitioners in remote communities was feasible with equivalent care delivered by GP, and was cost-saving.
Barnard, 2015 [37]	Primary care	RCTs and observational studies	Abortion procedures administered by mid-level providers (midwife or any other healthcare workers who has less training than doctors) compared to doctors.	Mid-level providers could be useful alternatives for medical or surgical abortions to reduce the number of deaths and the disability caused by unsafe abortion in resource limited setting. However, mid-level providers would need to be sufficiently trained and better monitoring of safety is required before widespread implementation.
Colvin, 2013 [41]	Primary care	Qualitative studies	Task shifting to and from midwife for midwifery services.	Task shifting may serve as a powerful means to address the crisis in human resources for maternal and newborn health, but requires careful planning, implementation and ongoing supervision and support to ensure optimal and safe impact.
Ogedegbe, 2014 [24]	Primary care in low-middle income countries	RCTs	Physician to non-physician substitution who provided patients with the WHO cardiovascular package protocol, which was a clinical decision and support tool for assessment and management of cardiovascular risk factor, lifestyle counselling, drug treatment protocol and referral pathways.	Small improvements in blood pressure among intervention group recipients.

RCT: randomised controlled trials.

### Outcomes of task shifting

####  

##### Task shifting to nurses

Task shifting to nurses reportedly improved access to care and was non-inferior on measures of clinical management such as cardiometabolic disease management, asthma control, antiretroviral therapy provision and hospitalisation rates. In the review by Martinez et al., the authors also found that more patients returned for consultations with nurses than physicians and had more face-to-face contact time leading to higher patient satisfaction scores [27]. There was also some evidence to suggest that task shifting to nurses was cost-effective [26]. These reviews also found that task shifting resulted in a better systolic and diastolic blood pressure among patients, with modest reductions between 3.73 and 5.91 mmHg in systolic and 1.48 and 2.54 mmHg in diastolic blood pressure [13,14,31]. Similarly, task shifting resulted in a slight improvement in HbA1c levels [13] (Supplementary Tables S1–S3). Other reported benefits include better outcomes in secondary prevention of cardiovascular diseases as well as better returning rates, especially among those receiving antiretroviral therapy [34].

##### Task shifting to pharmacists

Similar to task-shifting to nurses, the reviews reported that access to healthcare services was improved due to the diversification of modes of provision, where pharmacists took on the role as the first point of contact for minor ailments [27,36]. There was weak evidence to support pharmacist-led care for management of chronic diseases such as diabetes and hypertension. Task shifting was reported to result in a small improvement in HbA1c levels and blood pressure [39], with lower rates of onward referrals to primary care doctors [28,36].

##### Task shifting to other healthcare professionals

Task shifting to mid-level providers such as midwife was a viable alternative for medical and surgical abortions to reduce the number of deaths compared to unsafe abortion, especially in low-resource settings [37]. However, the review noted a need for proper planning and training given to midwife to ensure that these can be successfully implemented [41]. In another review that reported on blood pressure control, there appears to be some evidence to support task shifting [24].

### Cost and cost-effectiveness

From a societal perspective, Anthony et al., estimated that task shifting and role substitution to nurses for common minor ailments were cost-effective compared with GP care [26]. Two other reviews also found similar results and found that task-shifting allowed physicians to attend to more complex cases potentially resulting in personnel cost savings, and thus bring cost benefits to the health system [27,29]. Nevertheless, while pharmacist-led care was equally as effective as GP care, the evidence for cost-savings was unclear due to the limited number of studies [26,28].

### Key elements for successful implementation of task shift

**Figure 2.**
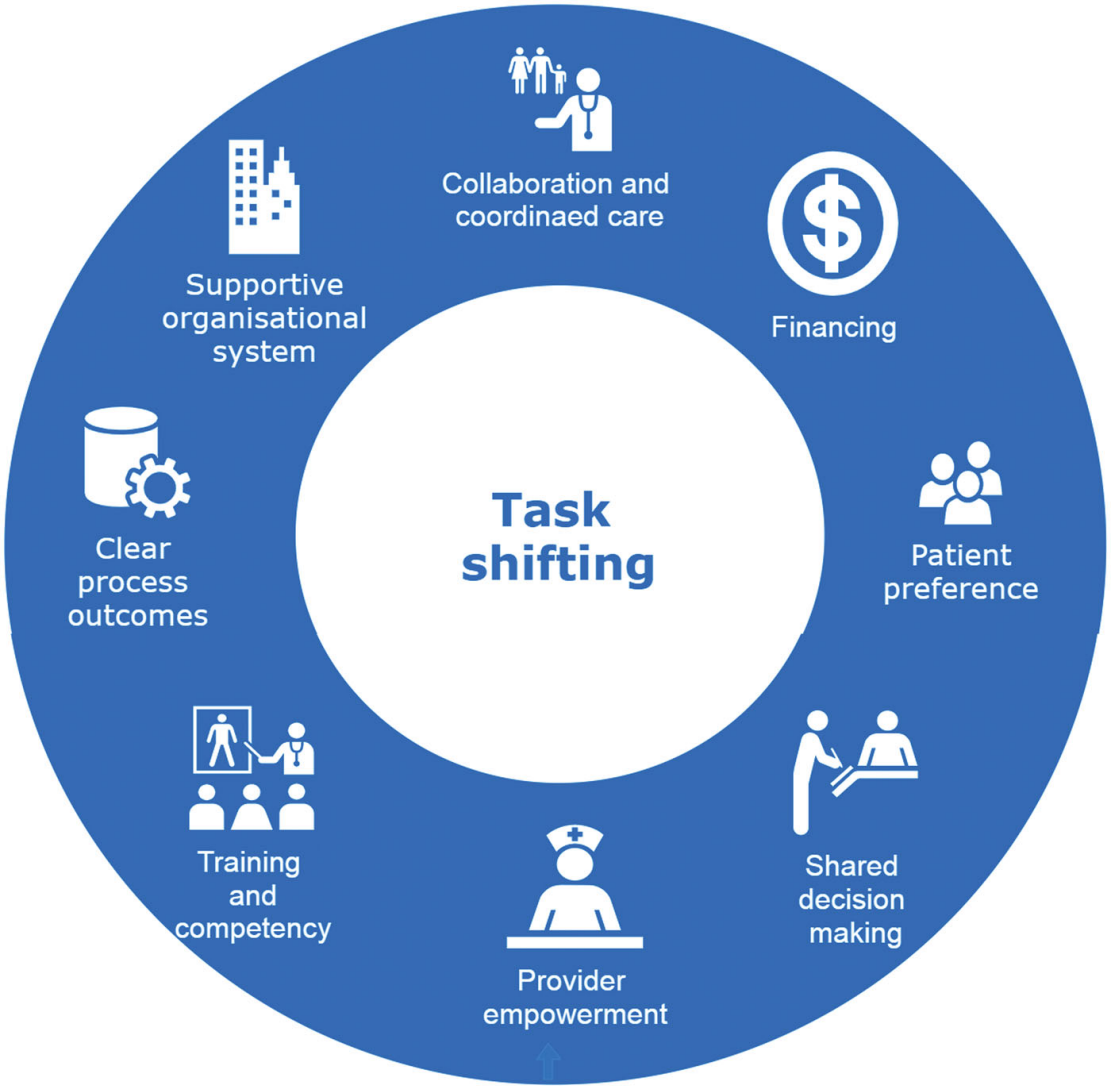
Elements for successful task shift.

The umbrella review identified several overarching themes, which could enhance or hinder the effectiveness of task shifting (Figure 2). A narrative synthesis is provided below.

### Collaboration and coordinated care

All reviews suggested that task shifting can enhance person-centred care in terms of patient-provider relationship. Two reviews also described the potential impact of task shifting as they allowed for greater use of healthcare resources and better access to healthcare [36,38]. Through task shifting, this allowed the limited number of specialists and physicians to practice in teams with other healthcare providers to reach to a larger population.

### Financing

One commonly identified barrier was the lack of clarity on the financial roles and responsibilities of the national and regional health authorities [26,38]. This was a major barrier since the choice of payment method can ultimately affect provider motivation [38]. This could be a potential source of conflict in healthcare systems which does not have universal healthcare coverage, especially when physician remuneration is derived from fee-for-service payments. In these situations, task shifting was perceived as a potential threat to the physicians’ income.

As task shifts were often used as a strategy to overcome the lack of manpower in health systems, there were limited studies that included any financial incentives in their implementation. In the two studies that examined the cost-effectiveness of task shift, it was noted that this strategy was the cost-effective means to provide care to patients in primary care [26,28].

### Patient preference and shared decision making

There has been a growing focus on empowerment of patients, as they can play a greater role in self-management. As such, patients and providers must be empowered to co-design their care guided by their goals and expectations. Thus, there is a need for all parties to be consulted and informed if the task shifts affect their care. The reviews reported that recipients of care generally have confidence in the competency of the other healthcare providers involved in routine care clinical work. However, they expressed a preference in receiving care from the physicians when the task was perceived to be more complex, requiring multidimensional care [15,35].

### Provider empowerment

Several reviews reported better job satisfaction among healthcare workers, due to the feeling of empowerment, ability to address local health needs and social recognition by the communities [35,38]. For example, the shifting of care of non-communicable diseases to nurses has been shown to have a positive impact on patients, with comparable and sometimes better outcomes.

### Training and competency

Several reviews described the importance of education and training activities to successfully implement at task shifts [34,35,37,40,41]. These studies described the need to enhance healthcare workers competencies, in particular when it involves role expansion. In addition, clinical supervision and support were identified as some of the important enablers to ensure the success of task shifts. Some examples include the training of nurse in nurse-led HIV/AIDS programme whereby the nurses underwent a practicum, training, supervision and mentorship with the physician prior to them providing consultation independently [34]. This is an important element in the successful implementation as the lack of support and training may result in them becoming apprehensive towards the new set of responsibilities they are expected to perform [35].

### Process outcome and supportive organisational system

When task shifting is implemented, a clear protocol on roles and responsibilities of each healthcare professional is needed to ensure the continuity of care and patient’s welfare is protected [27]. Support systems should be established to ensure that individuals with lower levels of training are supported with medical liability protection [40]. Several reviews identified in the current study have reported instances where task shifting led to role ambiguity and unclear boundaries between health professionals [15,35]. Increased workloads, lack of compensation and incentives are some issues identified during the implementation, which may impact the morale of staff [35,41].

### Quality of included reviews

Overall, seven reviews were graded as high quality (33.3%), three moderate (14.3%), four low (19.0%), and seven critically low quality (33.3%) using the AMSTAR-2 checklist. The main limitation of the reviews stem from the lack of description on reasons for exclusion of studies (*n* = 15, 71%). Another common limitation of most reviews was the lack of established protocols that were not published, limiting the study credibility. Most reviews had performed sufficiently comprehensive searches and provided a list of keywords used. Included reviews also assessed the risk of bias using appropriate checklists such as the Cochrane risk of bias tool or other appropriate checklists such as the Critical Appraisal Skills Programme checklist and Drummond checklist for economic evaluation. In all studies, all authors reported that data extraction was performed in duplicates. The most common non-critical flaw was the lack of reporting on funding sources for these reviews (Supplementary Table S4).

All reviews adhered to the PRISMA report guidelines regarding clear reporting of title, abstract, introduction, discussion and funding information (Supplementary Table S5). For reporting in methods, more than half of the reviews (*n* = 11) did not report about protocol and registration information, one review did not report the risk of bias in individual studies, and four reviews did not report on certainty of evidence (e.g. Grading of Recommendations, Assessment, Development and Evaluations (GRADE) assessment) to assess the confidence in the body of evidence for an outcome.

## Discussion

### Main findings

This detailed umbrella review synthesised evidence from existing systematic reviews and meta-analyses on task shift in primary care into one single document. Although similar reviews have been performed, these usually focussed on specific diseases such as HIV or did not specifically target primary care. This umbrella review found that task shifting to nurses can potentially lead to an increase in service provision, which could be cost-effective. Some of the commonly identified task shift includes provision of care and coordination of patients with chronic diseases as well as independent prescribing. Several reviews also reported patient satisfaction with task shifting [14,29,30], as they perceived they had received a better level of care as well as healthcare access. Recipients of care from several reviews reported they experienced better engagement due to the increased time spent during consultation as well as the possible removal of social and physical barriers that may exist with physicians [15,29].

A similar, albeit weaker association was noted when tasks were shifted to pharmacists. Evidence supports the roles of pharmacists for managing minor ailments, as there were fewer healthcare resource utilisation which could potentially be cost savings [28,36]. In addition, weak evidence was observed to support pharmacist-led services for medicines management in cardiovascular diseases [36]. For task shifting to other healthcare professionals such as midwifes, limited evidence exists to support its implementation.

To ensure the success of any task shifting exercise, establishment of good governance systems are crucial and these may include new legislation or amendment of existing law. Commonly identified barriers reported include poor staff coordination, low skills set, lack of support and recognition, as well as financial incentives. Other issues include the lack of access to patient records especially in community pharmacies. To prevent such governance issues, well thought-out policies and implementation strategies are needed prior to any large-scale implementation of task shifts, considering any cultural norms that may exist within a country or across districts.

### Methodological issues

While most of the evidence was derived from randomised controlled trials, some reviews included only observational studies [25,34,36], prone to residual confounding. Most of the included reviews pooled studies using random-effects model while several reviews performed the meta-analyses using a fixed-effects model [13,30,31,37], assuming that the studies are similar enough in terms of study design and settings. Given the wide variation in setting and how task shifting has been conducted, random-effects meta-analyses may be more appropriate [42]. Indeed, it would be ideal to reanalyse these meta-analyses to compare the results from fixed and random effects model; this was beyond the scope of the current umbrella review. In addition, we did not review the primary studies included in each of the meta-analysis that would have facilitated this. Another issue identified in the current study was that only one review [13] had examined the minor study effects or effects of publication bias visually using funnel plots. This was mainly due to the lack of primary studies in each review as most contained fewer than ten studies reporting on the same outcome, where Egger’s test is not recommended [43].

### Strengths and limitations

This study offers several strengths. Firstly, the study was conducted using a rigorous review process. However, as the umbrella review aims to provide a broad overview of the task shifting in primary care, it may not be directly applicable to all settings. Nevertheless, as primary care is the backbone of an effective health system, we believe the results could provide valuable insights for policymakers to improve service delivery [44]. To our best knowledge, this review is the first meta-epidemiological study on task shifting in primary care. Through this study, readers can better understand the research on task shifting conducted to date and identify gaps for future research. Finally, the methodological quality of all included studies was assessed using AMSTAR2 tool, and methodological rigour of the study was achieved by following PRISMA guidelines.

From our review, evaluation the impact of task shifting onto clinical outcomes and service delivery was not commonly reported. This warrants further research to better understand the sustainability and longitudinal effects of task shifting using standardised indicators such as hospitalisation rates, and other quality indicators such as readmission rates and mortality. The effective and successful implementation of task shifting is indeed one of the strategies that could help achieve the sustainable development goal 3 of good health and well-being, alongside strengthening healthcare systems [45]. The current study included variable quality reviews ranging from high to critically low quality based upon the AMSTAR-2 checklist. This mainly was because these studies did not justify the exclusion of studies in their review. Nevertheless, with the recent update in PRISMA guidelines [46], we believe that future studies will meet the necessary quality benchmarks as advocated using the AMSTAR-2 checklist.

We also found limited reviews that focussed and examined task shifting from low and middle-income countries. The lack of task shifting reviews from these countries could be due to the lack of studies conducted and reported in such settings. Another possible reason could be the exclusion of other paramedics such as community health workers from our review, which may have limited the number of studies. The latter has been extensively studied and examined in studies that focussed on HIV/AIDS care in low-middle income countries. Other limitations and caveats should also be considered in the interpretation of this study. We did not reanalyse any of the meta-analysis in our umbrella review nor review all the included primary studies, given that this was beyond the scope of our work. As such, we were unable to ascertain if the method of analysis used, including the use of fixed or random-effects model was appropriate in the reported analysis. Similarly some of the results of meta-analyses may have included studies from the same primary studies. We were also unable to conduct alternative tests, such as excess significant test, which attempts to detect reporting bias by comparing the number of studies that have formally substantial results against the number expected [47]. Finally, our review did not find any review that examined task shift to dieticians, paramedics and physiotherapists. This might be due to their specialised expertise, and more recognised roles in secondary or tertiary care settings than primary care [48].

### Implications for research and practice

Worldwide, there is increased interest in exploring the ability of allied health care professionals to extend their roles and scope of practice to overcome the lack of manpower, especially in primary care. This is even more important in pandemics where there is additional strain in healthcare resources and services. Results of the current review suggest a need to continuously engage with both the providers such as physicians as well as care recipients. As the change is not merely a delegation of work from physicians to other professionals but a new exploration of working together, this could potentially create problems to existing professional hierarchies. Thus, the rationale of change, mutual understanding and sharing a common goal should be emphasised across all parties to facilitate the sustainability of the reform [49].

Like most task shifting reviews that have been conducted to date, the breadth of task shifting models suggest that these results would need to be taken in light of the local health context and health system. For example, to provide healthcare services closer to home, especially to the urban poor, community clinics utilising available premises were established in Malaysia. The clinic could be managed by senior assistant medical officers and nurses, supervised by physicians at nearby primary care clinics. Without the support of local community and relevant stakeholders, this task shifting effort would not be possible to improve access to healthcare in vulnerable communities. Moving forward, combined effects between researchers, policymakers and other relevant stakeholders should be encouraged and promoted to ensure implemented task shifting are evaluated and reported. More programmatic research effort is needed to provide confirmatory evidence of the effectiveness of task shifting, access and quality to care as well as long-term financial implications.

## Conclusion

The current umbrella review found evidence supporting task shifting in primary care from physicians to allied healthcare workers such as pharmacists and nurses in primary care. Task shifted could include caring for patients, independent prescribing and health education for people with chronic diseases such as cardiometabolic and mental health issues. Task shifting appears to improve clinical outcomes among people with non-communicable diseases and mental health, with comparable quality of care.

## Supplementary Material

PRISMA 2009 Checklist

Supplementary Material
