# Supplementary material for: Task shifting in primary care to tackle healthcare worker shortages: An umbrella review
Source: Eur J Gen Pract. 2021 Aug 2;27(1):198–210. doi: 10.1080/13814788.2021.1954616 (PMC8330741; doi:10.1080/13814788.2021.1954616)
Supplement: Supplementary Material [file IGEN_A_1954616_SM1181.docx]

# Task shifting in primary care to tackle healthcare worker shortages: An umbrella review

# Supplementary material

Box S1: List of keywords used as a search strategy

1. Nurse* OR midwife OR midwifery OR midwives
2. paraprofessional* or paramedic or paramedics or paramedical worker* or paramedical personnel or allied health personnel or allied health worker*
3. pharmacy OR pharmacists OR pharmacies OR community pharmacy OR community pharmacy services OR community pharmacies OR pharmaceutical care OR pharmaceutical cares OR pharmaceutical services
4. dietitian* OR dietetic
5. physiotherapy OR physiotherapist
6. physician* OR doctor OR doctors OR general next practitioner* OR GP OR GPs OR family next practitioner*
7. primary care OR primary healthcare OR primary health care OR primary next practice* OR general next practice* OR family next practice* OR outpatient* OR ambulatory care OR community care OR community next health* OR community medicine OR home care
8. substitut* OR delegat* OR task* shift* OR change* responsabilit* OR extend*
9. task shift OR task shifting OR balance of care
10. nurse led OR nurse managed OR nurse run OR pharmacist led OR pharmacist managed OR pharmacist run OR paramedic led OR paramedic managed OR paramedic run OR allied health worker led OR allied health worker managed OR allied health worker run
11. systematic review OR meta-analysis
12. #1 OR #2 OR #3 OR #4 OR #5
13. #12 AND #6
14. #13 AND #7
15. #8 OR #9 OR #10
16. #15 AND #11
17. #16 AND #14

Sample search strategy from Pubmed.

| No | Search terms | Hits |
| --- | --- | --- |
| 1 | Nurse* OR midwife OR midwifery OR midwives | 471,041 |
| 2 | paraprofessional* or paramedic or paramedics or paramedical worker* or paramedical personnel or allied health personnel or allied health worker* | 63,021 |
| 3 | pharmacy OR pharmacists OR pharmacies OR community pharmacy OR community pharmacy services OR community pharmacies OR pharmaceutical care OR pharmaceutical cares OR pharmaceutical services | 581,678 |
| 4 | dietitian* OR dietetic | 31,320 |
| 5 | physiotherapy OR physiotherapist | 201,929 |
| 6 | physician* OR doctor OR doctors OR general next practitioner* OR GP OR GPs OR family next practitioner* | 901,156 |
| 7 | primary care OR primary healthcare OR primary health care OR primary next practice* OR general next practice* OR family next practice* OR outpatient* OR ambulatory care OR community care OR community next health* OR community medicine OR home care | 1,754,685 |
| 8 | substitut* OR delegat* OR task* shift* OR change* responsabilit* OR extend* | 877,351 |
| 9 | task shift OR task shifting OR balance of care | 50,368 |
| 10 | nurse led OR nurse managed OR nurse run OR pharmacist led OR pharmacist managed OR pharmacist run OR paramedic led OR paramedic managed OR paramedic run OR allied health worker led OR allied health worker managed OR allied health worker run | 234,206 |
| 11 | systematic review OR meta-analysis | 309,733 |
| 12 | #1 OR #2 OR #3 OR #4 OR #5 | 1,312,928 |
| 13 | #12 AND #6 | 113,935 |
| 14 | #13 AND #7 | 52,927 |
| 15 | #8 OR #9 OR #10 | 1,140,190 |
| 16 | #15 AND #16 | 26,497 |
| 17 | #16 AND #11 | 558 |

Table S1: Reported biochemical outcomes from meta-analyses on task shifts

| Study, year | Outcome | Effect size  (95% confidence interval)* | No of studies | No of participants | *I^2^* (%) |
| --- | --- | --- | --- | --- | --- |
| Laurant, 2018 | Diastolic blood pressure (mmHg) | MD: -2.54 (-4.57 to -0.52) | 2 | 562 | 0 |
| Nkansah, 2011 | Diastolic blood pressure (mmHg) | MD: -3.12 (-4.57 to -1.67) | 4 | 734 | 0 |
| Martinez-gonzalez 2014a | Diastolic blood pressure (mmHg) | MD: -1.48 (-3.05 to -0.09)^†^ | 4 | 836 | 38 |
| Laurant, 2018 | Systolic blood pressure (mmHg) | MD: -3.73 (-0.62 to -1.44) | 3 | 1,023 | 0 |
| Nkansah, 2011 | Systolic blood pressure (mmHg) | MD: -6.32 (-8.80 to -3.83) | 4 | 734 | 0 |
| Martinez-gonzalez 2014a | Systolic blood pressure (mmHg) | MD: -4.27 (-6.31 to -2.23) ^†^ | 5 | 1,344 | 0 |
| Weeks, 2016 | Systolic blood pressure (mmHg) | MD: -5.31 (-6.46 to -4.16)^†^  MD: -5.91 (-7.71 to -4.10)* | 12 | 4,229 | 50.4 |
| Laurant, 2018 | Total cholesterol (mmol/L) | MD: -0.15 (-0.3 to 0.02) | 2 | 702 | 0 |
| Martinez-gonzalez 2014a | Total cholesterol (mmol/L) | MD: -0.08 (-0.22 to 0.07)^†^ | 4 | 981 | 0 |
| Weeks, 2016 | Low-density lipoprotein, mmol/L | MD: -0.21 (-0.29 to -0.14)^†^  MD: -0.30 (-0.62 to 0.02)* | 7 | 1,469 | 93% |
| Martinez-gonzalez 2014a | HbA1c (%) | MD: 0.12 (-0.13 to 0.37)^†^ | 4 | 589 | 0 |
| Nkansah, 2011 | HbA1c (%) | MD: -0.75 (-1.41 to -0.09) | 2 | 260 | 24.8 |
| Weeks, 2016 | HbA1c (%) | MD: -0.62 (-0.85 to -0.38)^†^  MD: -0.62 (-0.85 to -0.38)* | 6 | 775 | 0 |
| Laurant, 2018 | HbA1c (%) | MD: 0.08 (-0.25 to 0.41) | 2 | 310 | 0 |

SMD: standardised mean difference; MD: mean difference; RR: relative risk

*random effects meta-analyses was used; ^†^ fixed effects meta-analyse was used

Table S2: Clinical outcomes from meta-analyses on task shifts

| Study, year | Outcome | Effect size*  (95% confidence interval) | No of studies | No of participants | *I^2^* (%) |
| --- | --- | --- | --- | --- | --- |
| Laurant, 2018 | Mortality | RR: 0.77 (0.57 to 1.03) | 8 | 36,529 | 0 |
| Martínez-González, 2014b | Mortality | RR: 0.89 (0.84 to 0.96) ^†^ | 10 | 14,652 | 0 |
| Kredo, 2014 | Death within 12 months for initiation and maintenance of ART (Cohort) | RR: 1.23 (1.14 to1.33) | 2 | 39 160 | 0 |
| Kredo, 2014 | Death within 12 months for initiation and maintenance of ART (RCTs) | RR: 0.96 (0.82 to1.12) | 1 | 2770 | - |
| Kredo, 2014 | Death within 12 months for maintenance of ART (Cohorts) | RR: 0.19 (0.05 to 0.78) | 1 | 2772 | - |
| Kredo, 2014 | Death within 12 months for maintenance of ART (RCTs) | RR: 0.89 (0.59 to 1.32) | 2 | 4332 | 0 |
| Kredo, 2014 | Death within 12 months for maintenance of ART in community (Cohorts) | RR: 1.44 (0.81 to 2.57) | 1 | 385 | - |
| Kredo, 2014 | Death within 12 months for maintenance of ART in community (RCTs) | RR: 1.00 (0.62 to 1.62) | 1 | 559 | - |
| Kredo, 2014 | Death or loss to follow-up within 12 months for maintenance of ART (Cohorts) | RR: 0.30 (0.17 to 0.54) | 1 | 2772 | - |
| Kredo, 2014 | Death or loss to follow-up within 12 months for maintenance of ART (RCTs) | RR: 1.1 (0.86 to 1.41) | 2 | 4332 | 0 |
| Kredo, 2014 | Death or lost to follow-up for maintenance of ART in community (Cohorts) | RR: 1.46 (0.98 to 2.17) | 1 | 385 | - |
| Kredo, 2014 | Death or lost to follow-up for maintenance of ART in community (RCTs) | RR: 0.93 (0.60 to 1.46) | 1 | 559 | - |
| Kredo, 2014 | Death or lost to follow-up within 12 months for initiation and maintenance of ART (Cohort) | RR: 0.72 (0.48 to 1.07) | 2 | 39 160 | 93 |
| Kredo, 2014 | Death or lost to follow-up within 12 months for initiation and maintenance of ART (RCTs) | RR: 0.89 (0.79 to 1.01) | 1 | 2770 | - |
| Laurant, 2018 | Hospital admission | RR: 1.04 (0.78 to 1.39) | 3 | 16,466 | 50.1 |
| Martínez-González, 2014b | Hospital admissions | RR: 0.76 (0.64 to 0.91) ^†^ | 5 | 3,890 | 7 |
| Laurant, 2018 | Hospital referral | RR: 0.90 (0.54 to 1.49) | 4 | 17,299 | 71.4 |
| van Ginneken, 2013 | Number of days spent in hospital at 1 year in treating depression in adults | MD: -1.79 (-3.59 to 0.01) | 1 | 124 | - |
| van Ginneken, 2013 | Number of days spent in hospital at 2 years in treating depression in adults | MD: -0.02 (-2.59 to 2.55) | 1 | 124 | - |
| van Ginneken, 2013 | Number of days spent on sick leave at 1 year in treating depression in adults | MD: -3.96 (-15.58 to 7.66) | 1 | 108 | - |
| van Ginneken, 2013 | Number of days spent on sick leave at 2 years in treating depression in adults | MD: 14.63 (-0.76 to 30.02) | 1 | 123 | - |
| Laurant, 2018 | Attendance at accident and emergency | RR: 1.00 (0.91 to 1.09) | 6 | 29,905 | 0 |
| Weeks, 2016 | Adherence | MD: 0.15 (0.00 to 0.30)^†^ | 4 | 700 | 38 |
| van Ginneken, 2013 | Adverse consequences in adults with alcohol-use disorders (RCTs) | RR: 0.77 (0.11 to 5.29) | 2 | 160 | 61 |
| van Ginneken, 2013 | Amount of alcohol consumed for adults with alcohol-use disorders within 3-6 months measured using the number of drinks/drinking day (in past week to 30 days) (RCTs) | MD: -1.68 (-2.79 to -0.57) | 2 | 167 | 0 |
| Laurant, 2018 | Attended return visit | RR: 1.19 (1.07 to 1.33) | 4 | 5,064 | 0 |
| van Ginneken, 2013 | Carer burden for dementia patients (RCTs) | SMD: -0.50 (-0.84 to -0.15) | 2 | 134 | 0 |
| van Ginneken, 2013 | Carer distress for dementia patients (RCTs) | SMD: -0.47 (-0.82 to -0.13) | 2 | 134 | 0 |
| van Ginneken, 2013 | Carer mental health status for dementia patients (RCTs) | SMD: -0.42 (-0.76 to -0.08) | 2 | 134 | 0 |
| van Ginneken, 2013 | Carer quality of life for dementia patients (RCTs) | MD: -0.37 (-0.92 to 0.17) | 1 | 53 | - |
| Barnard, 2015 | Complications: surgical abortion (observational studies). | RR: 1.38 (0.7 to2.72) | 3 | 13,715 | 43.5 |
| Barnard, 2015 | Complications: surgical abortion (RCTs). | RR: 0.99 (0.17 to5.7) ^†^ | 2 | 2,789 | 0 |
| Laurant, 2018 | Disease Activity Score | MD: 0.04 (-0.17 to 0.24) | 2 | NA | 0.5 |
| Barnard, 2015 | Failure/incomplete abortion and complications: surgical abortion (RCTs). | RR: 3.07(0.16 to 59.08) | 2 | 2,789 | 75.6 |
| Barnard, 2015 | Failure/incomplete medical abortion (observational) | RR: 1.09(0.63 to 1.88) | 1 | 1,164 | - |
| Barnard, 2015 | Failure/incomplete medical abortion (RCTs). | RR: 0.81(0.48 to 1.36)^†^ | 2 | 1,892 | 0 |
| Barnard, 2015 | Failure/incomplete surgical abortion (observational studies). | RR: 2.25(1.38 to 3.68)^†^ | 3 | 13,715 | 0 |
| Barnard, 2015 | Failure/incomplete surgical abortion (RCTs). | RR: 2.97(0.21 to 41.82) | 2 | 2,789 | 69.5 |
| van Ginneken, 2013 | Frequency of adverse events in treating depression in adults | RR: 0.85 (0.67 to 1.07) | 1 | 768 | - |
| van Ginneken, 2013 | Frequency of binge drinking in adults with alcohol-use disorders (RCTs) | MD: -0.50 (-1.14 to 0.14) | 1 | 92 | - |
| van Ginneken, 2013 | Functional ability of dementia patient (RCTs) | MD: -0.24 (-0.67 to 0.20) | 1 | 81 | - |
| van Ginneken, 2013 | Functional impairment in PTSD children within 11 months post teacher/LHW-led interventions | SMD: -0.69 (-1.25 to -0.14) | 1 | 53 | - |
| van Ginneken, 2013 | Functional impairment in PTSD children within 6 months post LHW-led classroom-based interventions | MD: -0.81 (-1.48 to -0.13) | 3 | 1092 | 7 |
| van Ginneken, 2013 | Functional impairment in PTSD children within 6 months post teacher/LHW-led interventions | SMD: -0.61 (-1.13 to -0.08) | 2 | 220 | 18 |
| van Ginneken, 2013 | Functional impairment/disability in common mental disorders within 6 months post intervention (RCTs) | SMD: -0.33 (-0.80 to 0.13) | 4 | 1243 | 90 |
| van Ginneken, 2013 | Functional impairment/disability in common mental disorders within 8 months post intervention (RCTs) | SMD: -0.56 (-0.70 to - 0.42) | 1 | 798 | - |
| Laurant, 2018 | Investigations | RR: 0.95 (0.59 to 1.51) | 4 | 3,654 | 76.2 |
| Laurant, 2018 | Length of consultation | SMD: 0.38 (0.22 to 0.54) | 4 | 5,848 | 89.7 |
| Kredo, 2014 | Lost to follow-up for maintenance of ART in community (Cohorts) | RR: 1.49 (0.81 to 2.74) | 1 | 385 | - |
| Kredo, 2014 | Lost to follow-up for maintenance of ART in community (RCTs) | RR: 0.52 (0.12 to 2.30) | 1 | 559 | - |
| Kredo, 2014 | Lost to follow-up within 12 months for initiation and maintenance of ART (Cohorts) | RR: 0.30 (0.05 to 1.94) | 2 | 39 156 | 98 |
| Kredo, 2014 | Lost to follow-up within 12 months for initiation and maintenance of ART (RCTs) | RR: 0.73 (0.55 to0.97) | 1 | 2770 | - |
| Kredo, 2014 | Lost to follow-up within 12 months for maintenance of ART (Cohorts) | RR: 0.34 (0.18 to 0.66) | 1 | 2772 | - |
| Kredo, 2014 | Lost to follow-up within 12 months for maintenance of ART (RCTs) | RR: 1.27 (0.92 to 1.77) | 2 | 4332 | 0 |
| Laurant, 2018 | Pain | MD: 0.76 (-3.85 to 5.38) | 2 | NA | 0 |
| Laurant, 2018 | Physical function | RR: 1.03 (0.98 to 109) | 3 | 3,549 | 61.7 |
| Laurant, 2018 | Prescription ordered | RR: 0.99 (0.95 to 1.03) | 4 | 5,702 | 4.7 |
| van Ginneken, 2013 | Prevalence of depression in adults (completers) within 6 months post intervention (RCTs) | RR: 0.30 (0.14 to 0.64) | 3 | 1082 | 81 |
| van Ginneken, 2013 | Prevalence of post-traumatic stress disorder (PTSD) within 6 months post LHW-led narrative exposure therapy (RCT and NRCT) | RR: 0.48 (0.27 to 0.85) | 1 | 62 | - |
| van Ginneken, 2013 | Prevalence of post-traumatic stress disorder within 6 months post LHW-led trauma counselling (RCT and NRCT) | RR: 0.55 (0.33 to 0.93) | 1 | 65 | - |
| van Ginneken, 2013 | Road traffic accidents in adults with alcohol-use disorders (RCTs) | RR: 0.36 (0.12 to 1.08) | 1 | 92 | - |
| Laurant, 2018 | Scheduled return visits | RR: 1.31 (0.89 to 1.94) | 3 | 3,934 | 85.6 |
| van Ginneken, 2013 | Severity of anxiety symptoms in PTSD children within 6 months post interventions | MD: -0.34 (-0.75 to 0.07) | 3 | 1092 | 18 |
| van Ginneken, 2013 | Severity of common mental disorder symptoms (includes anxiety and depression) within 1 years post intervention (RCTs) | SMD: -0.47 (-0.60 to -0.34) | 2 | 923 | 0 |
| van Ginneken, 2013 | Severity of common mental disorder symptoms (includes anxiety and depression) within 6 months post intervention (RCTs) | SMD: -0.75 (-1.29 to -0.21) | 6 | 1470 | 94 |
| van Ginneken, 2013 | Severity of dementia patients’ behavioural problem within 6 months measured using the behavioural symptom scale (RCTs) | SMD: -0.26 (-0.60 to 0.08) | 2 | 134 | 0 |
| van Ginneken, 2013 | Severity of depression in adult patients (0-56 days) measured using HDRS | MD: -0.90 (-1.20 to -0.60) | 1 | 768 | - |
| van Ginneken, 2013 | Severity of depression in PTSD patients with 2 weeks post LHW-led workshop with psychoeducation | MD: -0.07 (-0.36 to 0.22) | 1 | 76 | - |
| van Ginneken, 2013 | Severity of depression in PTSD patients with 2 weeks post LHW-led workshop without psychoeducation | MD: -0.14 (-0.42 to 0.14) | 1 | 75 | - |
| van Ginneken, 2013 | Severity of depressive symptoms in PTSD children within 6 months post classroom-based LHW interventions (MCDs) | MD: -0.18 (-0.33 to -0.03) | 3 | 1092 | 0 |
| van Ginneken, 2013 | Severity of depressive symptoms in PTSD children within 6 months post teacher/LHW-led interventions (SMDs) | SMD: -0.23 (0.45 to -0.02) | 4 | 504 | 17 |
| van Ginneken, 2013 | Severity of PTSD symptoms within 11 months teacher/LHW-led interventions (children) (RCTs) | SMD: -0.45 (-0.99 to 0.10) | 1 | 53 | - |
| van Ginneken, 2013 | Severity of PTSD symptoms within 11 months teacher/LHW-led interventions (children) (RCTs) | SMD: -0.04 (-0.59 to 0.52) | 1 | 51 | - |
| van Ginneken, 2013 | Severity of PTSD symptoms within 2 weeks post NSHW-led psychological interventions (RCT and NRCT) | SMD: -0.44 (-0.90 to 0.02) | 1 | 75 | - |
| van Ginneken, 2013 | Severity of PTSD symptoms within 5 months teacher/LHW-led interventions (children) (RCTs) | SMD: -0.12 (-0.67 to 0.44) | 1 | 51 | - |
| van Ginneken, 2013 | Severity of PTSD symptoms within 6 months in teacher/LHW-led interventions (children) (RCTs) | SMD: -0.89 (-1.49 to -0.30) | 3 | 298 | 78 |
| van Ginneken, 2013 | Severity of PTSD symptoms within 6 months post classroom-based LHW interventions (boys/girls) (RCTs) | MD: 1.40 (-1.58 to 4.37) | 1 | 399 | - |
| van Ginneken, 2013 | Severity of PTSD symptoms within 6 months post classroom-based LHW interventions (MCDs) (children)(RCTs) | MD: -0.56 (-2.82 to 1.70) | 3 | 1090 | 82 |
| van Ginneken, 2013 | Severity of PTSD symptoms within 6 months post classroom-based LHW interventions in boys (RCTs) | MD: 0.0 (-2.02 to 2.02) | 1 | 245 | - |
| van Ginneken, 2013 | Severity of PTSD symptoms within 6 months post classroom-based LHW interventions in girls (RCTs) | MD: 3.05 (0.39 to 5.71) | 1 | 154 | - |
| van Ginneken, 2013 | Severity of PTSD symptoms within 6 months post LHW-led counselling with PTSD psychoeducation (RCT and NRCT) | SMD: -0.36 (-0.67 to -0.05) | 3 | 223 | 22 |
| van Ginneken, 2013 | Severity of PTSD symptoms within 6 months post narrative exposure therapy (RCT and NRCT) | SMD: -0.55 (-1.08 to -0.03) | 1 | 75 | - |
| van Ginneken, 2013 | Severity of symptoms in treating maternal depression at 1 year post intervention (RCTs) | SMD: -0.41 (-0.76 to -0.06) | 1 | 125 | - |
| van Ginneken, 2013 | Severity of symptoms in treating maternal depression within 3 months post intervention (RCTs) | SMD: -0.50 (-0.63 to -0.36) | 2 | 858 | 0 |
| van Ginneken, 2013 | Severity of symptoms of perinatal depression (0-12 months) measured using various depression rating scales | SMD: -0.42 (-0.58 to -0.26) | 4 | 1213 | 29 |
| Barnard, 2015 | Total failures/incomplete abortion and complications: surgical abortion (observational). | RR: 1.36 (0.86 to2.14) | 4 | 16,173 | 61.2 |
| van Ginneken, 2013 | Withdrawal symptoms in adults with alcohol-use disorders (RCTs) | RR: 2.67 (0.29 to 24.37) | 1 | 68 | - |

SMD: standardised mean difference; MD: mean difference; RR: relative risk

*random effects meta-analyses was used; ^†^ fixed effects meta-analyse was used

Table S3: Patient reported outcomes from meta-analyses on task shifts

| Study, year | Outcome | Effect size*  (95% confidence interval) | No of studies | No of participants | *I^2^* (%) |
| --- | --- | --- | --- | --- | --- |
| Weeks, 2016 | Health-related quality of life, SF-12/36 - Mental component | MD: 0.58 (-0.40, 1.55) ^†^ | 8 | 4,631 | 66 |
| Weeks, 2016 | Health-related quality of life, SF-12/36 - Physical component | MD: 1.17 (0.16, 2.17) ^†^ | 8 | 4,631 | 17 |
| Laurant, 2018 | Quality of life | SMD: 0.16 (0.00, 0.31) | 6 | 16,002 | 85.3 |
| van Ginneken, 2013 | Quality of life of dementia patient | MD: -0.43 (-0.98, 0.12) | 1 | 53 | - |
| Laurant, 2018 | Patient satisfaction | SMD: 0.08 (0.01, 0.15) | 7 | 16,993 | 55.9 |
| Martínez-González, 2014b | Patient satisfaction | SMD: 0.18 (0.13, 0.23)^†^ | 7 | 5,821 | 91 |

SMD: standardised mean

*random effects meta-analyses was used; ^†^ fixed effects meta-analyse was used

Table S4: Quality rating of included reviews based upon the AMSTAR 2 tool

| Author, Year | Q1 | Q2 | Q3 | Q4 | Q5 | Q6 | Q7 | Q8 | Q9 | Q10 | Q11 | Q12 | Q13 | Q14 | Q15 | Q16 | Overall confidence rating |
| --- | --- | --- | --- | --- | --- | --- | --- | --- | --- | --- | --- | --- | --- | --- | --- | --- | --- |
| Anthony 2019 | Yes | No | NA | Yes | Yes | No | Yes | Yes | Yes | No | NA | NA | Yes | Yes | NA | Yes | Low |
| Barnard 2015 | Yes | Yes | No | Yes | Yes | Yes | Yes | Yes | Yes | No | Yes | NA | Yes | Yes | NA | Yes | Moderate |
| Chapman 2004 | Yes | No | No | Yes | No | No | No | Yes | Yes | No | NA | NA | No | NA | NA | Yes | Very low |
| Colvin 2013 | Yes | No | Yes | Yes | Yes | No | No | Yes | Yes | No | NA | NA | No | NA | NA | Yes | Very low |
| Jebara, 2018 | Yes | Yes | No | Yes | Yes | Yes | No | Yes | No | No | NA | NA | No | No | NA | Yes | Very low |
| Karimi-Shahanjarini 2019 | Yes | Yes | No | Yes | Yes | No | Yes | Yes | Yes | No | NA | NA | Yes | NA | NA | Yes | Moderate |
| Kredo, 2014 | Yes | Yes | Yes | Yes | Yes | Yes | Yes | Yes | Yes | No | Yes | Yes | Yes | Yes | Yes | Yes | High |
| Laurant, 2018 | Yes | Yes | Yes | Yes | Yes | Yes | Yes | Yes | Yes | No | Yes | Yes | Yes | Yes | Yes | Yes | High |
| Martinez-gonzalez 2014a | Yes | No | No | Yes | Yes | Yes | Yes | Yes | Yes | Yes | Yes | No | Yes | Yes | NA | Yes | Low |
| Martinez-gonzalez 2014b | Yes | Yes | Yes | Yes | Yes | Yes | Yes | Yes | Yes | Yes | Yes | Yes | Yes | Yes | Yes | Yes | High |
| Martinez-gonzalez 2015a | Yes | Yes | Yes | Yes | Yes | Yes | Yes | Yes | Yes | Yes | NA | NA | Yes | NA | NA | Yes | High |
| Martinez-gonzalez 2015b | Yes | Yes | Yes | Yes | Yes | Yes | Yes | Yes | Yes | Yes | NA | NA | No | No | NA | Yes | Low |
| Martinez-gonzalez 2015c | Yes | Yes | Yes | Yes | Yes | Yes | No | Yes | Yes | Yes | No | Yes | Yes | Yes | Yes | Yes | Very low |
| Nkansah, 2010 | Yes | Yes | Yes | Yes | Yes | Yes | Yes | Yes | Yes | No | Yes | No | Yes | No | No | Yes | Low |
| Ogedegbe, 2014 | Yes | Yes | Yes | Yes | Yes | Yes | Yes | Yes | Yes | No | NA | NA | Yes | NA | NA | Yes | High |
| Paudyal, 2013 | Yes | Yes | Yes | Yes | Yes | Yes | Yes | No | Yes | No | NA | NA | Yes | NA | NA | Yes | Moderate |
| Rashid, 2010 | Yes | No | Yes | Yes | Yes | Yes | No | Yes | No | No | NA | NA | No | NA | NA | Yes | Very low |
| van Ginneken, 2013 | Yes | Yes | Yes | Yes | Yes | Yes | Yes | Yes | Yes | Yes | Yes | Yes | Yes | Yes | Yes | Yes | High |
| Weeks 2016 | Yes | Yes | No | Yes | Yes | Yes | Yes | Yes | Yes | Yes | Yes | Yes | Yes | Yes | Yes | Yes | High |
| Whiteford, 2016 | Yes | No | Yes | Yes | Yes | Yes | Yes | Yes | No | No | NA | NA | No | NA | NA | No | Very low |
| Zhou, 2019 | Yes | No | No | Yes | No | Yes | No | No | No | No | NA | NA | No | NA | NA | Yes | Very low |

NA – not applicable

**Domains of the AMSTAR 2 tool**

1. Did the research questions and inclusion criteria for the review include the components of PICO?
2. Did the report of the review contain an explicit statement that the review methods were established prior to the conduct of the review and did the report justify any significant deviations from the protocol?
3. Did the review authors explain their selection of the study designs for inclusion in the review?
4. Did the review authors use a comprehensive literature search strategy?
5. Did the review authors perform study selection in duplicate?
6. Did the review authors perform data extraction in duplicate?
7. Did the review authors provide a list of excluded studies and justify the exclusions?
8. Did the review authors describe the included studies in adequate detail?
9. Did the review authors use a satisfactory technique for assessing the risk of bias (RoB) in individual studies that were included in the review?
10. Did the review authors report on the sources of funding for the studies included in the review?
11. If meta-analysis was performed did the review authors use appropriate methods for statistical combination of results?
12. If meta-analysis was performed, did the review authors assess the potential impact of RoB in individual studies on the results of the meta-analysis or other evidence synthesis?
13. Did the review authors account for RoB in individual studies when interpreting/ discussing the results of the review?
14. Did the review authors provide a satisfactory explanation for, and discussion of, any heterogeneity observed in the results of the review?
15. If they performed quantitative synthesis did the review authors carry out an adequate investigation of publication bias (small study bias) and discuss its likely impact on the results of the review?
16. Did the review authors report any potential sources of conflict of interest, including any funding they received for conducting the review?

Table S5: Quality of reporting of included reviews based upon the Preferred Reporting Items for Systematic Reviews and Meta-Analyses (PRISMA).

| Author, Year | Title  #1 | Abstract  #2 | Introduction  #3-4 | Methods  #5-11,13,14,16 | Methods  #12 (Risk of bias in individual studies) | Methods  #15 (Certainty of evidence) | Results  #17-23 | Discussion  #24-26 | Funding  #27 |
| --- | --- | --- | --- | --- | --- | --- | --- | --- | --- |
| Anthony 2019 | Yes | Yes | Yes | Yes except Protocol and registration | Yes | Yes | Yes | Yes | Yes |
| Barnard 2015 | Yes | Yes | Yes | Yes | Yes | Yes | Yes | Yes | Yes |
| Chapman 2004 | Yes | Yes | Yes | Yes except Protocol and registration | No | Yes | Yes except Risk of bias in individual studies | Yes | Yes |
| Colvin 2013 | Yes | Yes | Yes | Yes except Protocol and registration | Yes | Yes | Yes | Yes | Yes |
| Jebara, 2018 | Yes | Yes | Yes | Yes | Yes | Yes | Yes | Yes | Yes |
| Karimi-Shahanjarini 2019 | Yes | Yes | Yes | Yes | Yes | Yes | Yes | Yes | Yes |
| Kredo, 2014 | Yes | Yes | Yes | Yes | Yes | Yes | Yes | Yes | Yes |
| Laurant, 2018 | Yes | Yes | Yes | Yes | Yes | Yes | Yes | Yes | Yes |
| Martinez-gonzalez 2014a | Yes | Yes | Yes | Yes except Protocol and registration | Yes | No | Yes except Certainty of evidence | Yes | Yes |
| Martinez-gonzalez 2014b | Yes | Yes | Yes | Yes except Protocol and registration | Yes | No | Yes except Certainty of evidence | Yes | Yes |
| Martinez-gonzalez 2015a | Yes | Yes | Yes | Yes except Protocol and registration | Yes | No | Yes except Certainty of evidence | Yes | Yes |
| Martinez-gonzalez 2015b | Yes | Yes | Yes | Yes except Protocol and registration | Yes | Yes | Yes | Yes | Yes |
| Martinez-gonzalez 2015c | Yes | Yes | Yes | Yes except Protocol and registration | Yes | No | Yes except Certainty of evidence | Yes | Yes |
| Nkansah, 2010 | Yes | Yes | Yes | Yes | Yes | Yes | Yes | Yes | Yes |
| Ogedegbe, 2014 | Yes | Yes | Yes | Yes except Protocol and registration | Yes | Yes | Yes | Yes | Yes |
| Paudyal, 2013 | Yes | Yes | Yes | Yes | Yes | Yes | Yes | Yes | Yes |
| Rashid, 2010 | Yes | Yes | Yes | Yes except Protocol and registration | Yes | Yes | Yes | Yes | Yes |
| van Ginneken, 2013 | Yes | Yes | Yes | Yes | Yes | Yes | Yes | Yes | Yes |
| Weeks 2016 | Yes | Yes | Yes | Yes | Yes | Yes | Yes | Yes | Yes |
| Whiteford, 2016 | Yes | Yes | Yes | Yes | Yes | Yes | Yes | Yes | Yes |
| Zhou, 2019 | Yes | Yes | Yes | Yes except Protocol and registration | Yes | Yes | Yes | Yes | Yes |
| #PRISMA checklist item number | | | | | | | | | |
